# Self-awareness of hepatitis C infection in the United States: A cross-sectional study based on the National Health Nutrition and Examination Survey

**DOI:** 10.1371/journal.pone.0293315

**Published:** 2023-10-24

**Authors:** Karthik Gnanapandithan, Maged P. Ghali

**Affiliations:** 1 Division of Hospital Internal Medicine, Mayo Clinic, Jacksonville, Florida, United States of America; 2 Department of Gastroenterology and Hepatology, University of Florida Health, Jacksonville, Florida, United States of America; Centers for Disease Control and Prevention, UNITED STATES

## Abstract

Hepatitis C virus (HCV) is a global health issue with an estimated prevalence of 2.4 to 3 million people in the US and 58 million worldwide. Previous reports from the US have shown that close to half of those with the infection are unaware of their status. Although the current therapy for HCV is very effective, the primary barrier has been the inability to diagnose a large fraction of those infected. We studied public awareness of HCV in the US using National Health Nutrition and Examination Survey data from 2013 to 2020. Our aim was to measure awareness of infection in individuals with HCV and identify possible barriers to diagnosis. In total, 206 individuals with HCV were included in the weighted analysis. The weighted awareness of infection was 60.1%, suggesting that over 0.8 million are unaware nationally. Awareness was significantly low in the Mexican American and Asian populations. Non-US citizens and non–US-born individuals also had poor awareness. The transaminases were more elevated in those unaware of the infection, suggesting their higher risk of liver fibrosis. Although the proportion of infected people aware of their illness has been rising, over 0.8 million are still unaware of their infection and their risk of liver damage. We believe policy measures focused on further intense screening and educational campaigns, particularly in high-risk groups, are vital in realizing the World Health Organization’s goal of eliminating HCV as a global health threat.

## Background

Hepatitis C virus (HCV) is the most common blood-borne pathogen in the US [1] It is one of the leading etiologies of liver-related mortality and morbidity. It remains a global concern despite the combined efforts of the World Health Organization (WHO) and other health organizations. In 2016, the WHO set a target to eliminate HCV and hepatitis B virus as a public health threat by 2030. However, this requires that 90% of individuals with active HCV infection be diagnosed, 80% of these be treated with the intent to cure, and other measures be initiated to reduce the incidence of HCV in high-risk populations [2,3]. In 2021, the interim WHO guidelines [4] require an absolute annual incidence of ≤5 per 100,000 in the general population and ≤2 per 100 among people who inject drugs and an HCV-related yearly mortality of ≤2 per 100,000 persons for countries seeking validation towards HCV elimination. The development of direct-acting antivirals (DAAs) for HCV infection is a major advancement in medical science, improving the cure rate of HCV infection to more than 90% [5–7]. Still, extensive screening and treatment implementation programs are required for DAA therapy to reach its full potential at the population level. Surveillance data from the Centers for Disease Control and Prevention (CDC) show that after a decline in the number of acute HCV cases between 1990 to 2005 and a leveling off from 2006 to 2010, there has been an increase from 2010 to 2020 [8] The estimated number of acute HCV infections in 2020 was 66,700. The number of acute cases and deaths related to HCV increased in 2020 compared to 2019 [8] It is the second leading cause of liver transplant, closely following nonalcoholic fatty liver disease [9] The burden of HCV-associated disease is predicted to increase during the next 10 to 20 years as the infected cohort ages, and complications related to liver disease are more likely to occur. HCV infection among younger adults has risen due to the opioid and infectious diseases syndemic. The 2020 revised guidelines [10] recommended testing at least once for everyone aged 18 and older and pregnant women during every pregnancy.

Most acute HCV infections tend to be asymptomatic, which can lead to underdiagnosis. The SARS-CoV-2 pandemic has also affected screening and treatment programs over the last 2 to 3 years. Above all, the lack of awareness of infection is a major obstacle to the effectiveness of HCV elimination programs. It has been shown that almost 45% of those living with HCV were unaware of their infection status [11], and this has not changed much with time [12] In the US, the prevalence of HCV is disproportionate in some populations [13,14] A thorough report on the trends of self-awareness of HCV infection over the years and the difference in awareness between various demographic groups is vital to progress toward reducing the prevalence and transmission of HCV [15]. Apart from a better understanding of HCV awareness disparities, this can facilitate improved resource allotment and public health interventions.

## Methods

### Study population

Historically, the National Health and Nutrition Examination Survey (NHANES) has been used to derive the prevalence of HCV in the US [16] By testing a nationally representative sample of the US population, NHANES provides a reliable estimate of the age-specific prevalence of HCV needed to evaluate the effectiveness of preventive strategies. Apart from the data on anti-HCV serology and HCV RNA required for diagnosis, it also contains information collected regarding individuals’ self-awareness regarding their HCV infection. NHANES is conducted by the National Center for Health Statistics of the CDC every two years. It is an ongoing cross-sectional database composed of interviews, physical examination, and laboratory data from a series of multistage, stratified, clustered sample. It is representative of the US population, with an intentional oversampling of African American and Hispanic populations. A limitation pertinent to NHANES is that the institutionalized and homeless populations are not represented in the sample. The National Center for Health Statistics Research Ethics Review Board approves the NHANES study, and all participants provide written informed consent. We used data from the following survey cycles: 2013–2014, 2015–2016, 2017–2018, and 2019–2020. NHANES had to stop operations in March 2020 due to the COVID-19 pandemic and the 2019–2020 cycle could not be completed. The collected data were combined with the 2017–2018 cycle to create a nationally representative January 2017 to March 2020 pre-pandemic cycle. Adults 20 years or older were included in the study, as complete interviews and laboratory data were available in this age group.

### Direct interview data

The medical conditions section of the NHANES interview provides self-reported personal interview data on a broad range of health conditions. Demographic data is collected from the participants in person. It provides information on several variables, including age, race and ethnicity, and sex (recorded as male and female). The hepatitis questionnaire provides respondent-level interview data on previous diagnoses of HCV by a health professional. Interviews are conducted at home by trained personnel with the help of a computer-assisted system. Other data collected include level of education, annual family income, access to health care, and type of health insurance, if any. All data are reviewed for completeness, consistency, and illogical values.

### Laboratory methods

**Fig 1:**
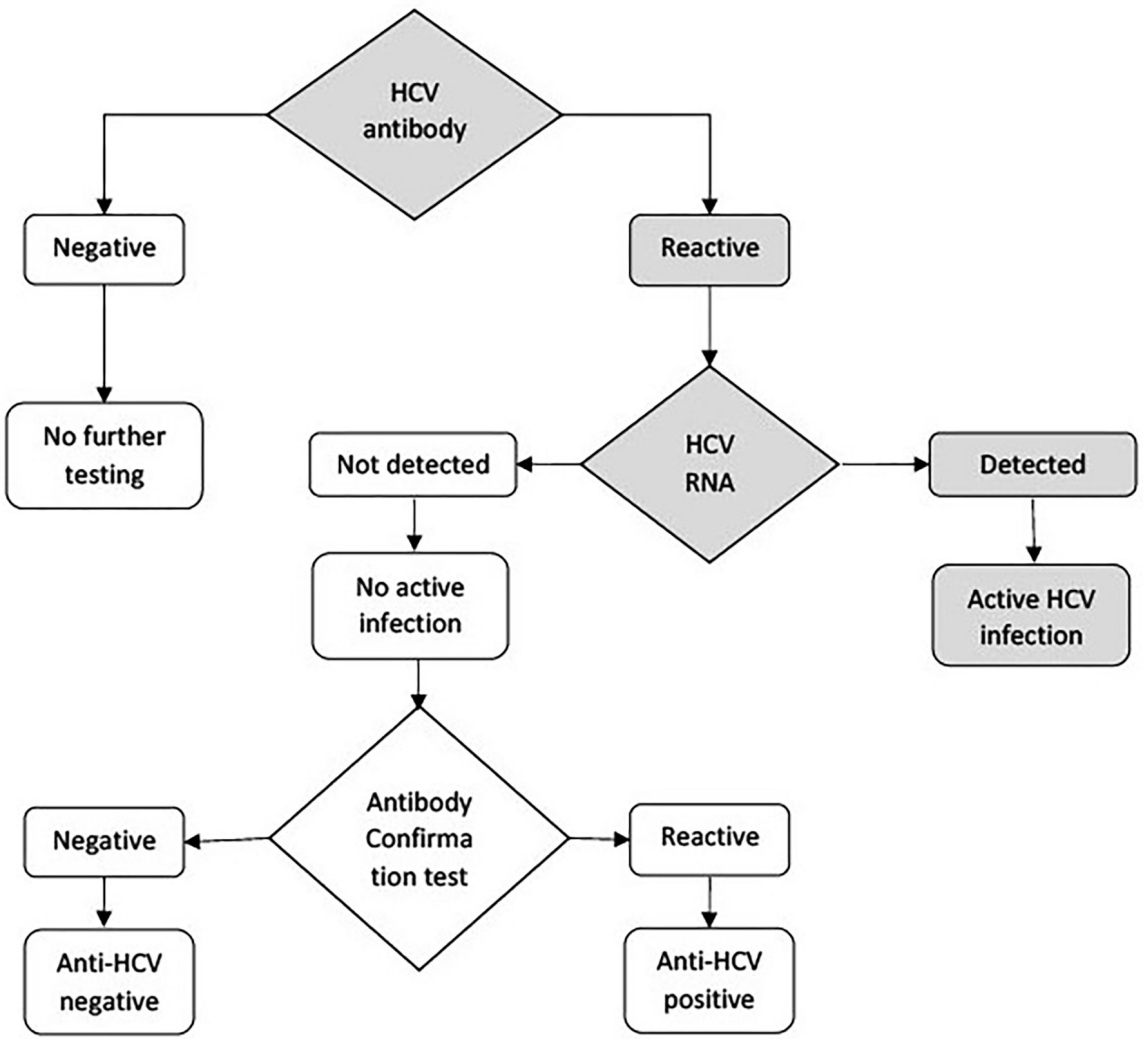
Hepatitis C Virus (HCV) testing algorithm, national health and nutrition examination survey 2013–2020.

There was a change in the NHANES HCV testing algorithm in 2013 per CDC recommendation [17] (Fig 1). HCV antibody testing is initially performed on all samples. All samples that are reactive to the antibody screening are then tested for RNA. The COBAS AMPLICOR HCV monitor test, version 2.0 (Hoffmann-La Roche Ltd), is a nucleic acid amplification test that quantifies HCV RNA in the serum samples. The antibody-positive samples that subsequently test RNA positive are considered to have active HCV infection. All RNA-positive samples are tested for genotype. The RNA-negative samples are tested with an antibody confirmation test using the third-generation manual 16-hour sample incubation test procedure. Samples with a positive antibody confirmation test result are reported as anti-HCV positive, and those with a negative antibody confirmation test result are reported as negative. The VERSANT HCV Genotype 2.0 Assay (Siemens Medical Solutions USA, Inc), a line probe assay, identifies HCV genotypes 1 to 6 in the serum samples. Subtype information was available in most cases.

### Statistical analysis

As NHANES is representative rather than simple random sampling, analysis of NHANES data must utilize sample weights to account for the unequal selection probabilities and missing data to estimate the actual prevalence of HCV in the US population (reported as weighted prevalence). Data analyses were carried out using SAS, version 9.4 (SAS Institute Inc). Data from different files were merged using the unique sequence number given to each participant. Overall weighted self-awareness and self-awareness across different subgroups (age, race and ethnicity, sex, level of education, and annual family income) were obtained using PROC SURVEYFREQ. Age was grouped into four categories (20–35, 36–50, 51–65, and >65 years), and ethnicity into five categories (Hispanic, non-Hispanic White, non-Hispanic Black, Asian, and Other). The other parameters considered were educational level, annual family income, insurance, access to health care, and citizenship. Logistic regression (PROCSURVEYLOGISTIC) was used to determine the odds of better self-awareness of HCV infection across the different groups and determine any improvement between the years (2017–2020 and 2015–2016 compared to 2013–2014). Appropriate subsample weights in accordance with NHANES guidelines were used in each of the above steps. PROCSURVERYMEANS was used to determine the mean liver function tests (LFTs) for participants with and without active HCV infection. The difference in LFTs was also studied between the participants who were aware of their infection and those who were unaware. Linear regression output determined if these were statistically significant. The level of significance was set at 5%.

## Results

There were 206 adult participants with HCV infection (positive HCV RNA) in the NHANES between January 2013 and March 2020. The weighted awareness of infection in the US population was 60.1% over the study period. This corresponds to approximately 0.84 million adults in the US with HCV who are unaware of their infection. Table 1 shows the distribution of disease awareness across different groups. Mexican American and Asian populations had much lower awareness at 39.8% and 13.4%, respectively. The odds of awareness of infection were 0.33 (0.15–0.72) in Mexican American participants and 0.07 (0.0007–0.79) in Asian participants compared to non-Hispanic whites. Disease awareness was also considerably low in the Hispanic and Black populations. The difference persisted even after correcting for age, socioeconomic status, and access to health care. Knowledge of the disease was significantly lower in non-US citizens (p<0.001) and non–US-born participants (odds 0.19[0.06–0.55]). There was no significant difference in awareness based on sex, level of education, annual family income, or insurance. The proportion of those aware was lower in the 36 to 50-year age cohort, although this was insignificant.

**Table 1:** Self-awareness of HCV infection (RNA positivity), 2013–2020 (N = 206).

	HCV awareness, weighted % (No.) (n = 206)	Adjusted OR (95% CI)
Age group, years 20–35 36–50 51–65 >65	58.3% (5/11) 46.1% (17/38) 67.7% (67/118) 58.6% (19/39)	1.64 (0.26–9.97) REF 2.45 (0.78–7.65) 1.66 (0.47–5.80)
Sex Male Female	62.1% (75/145) 58.5% (33/61)	REF 0.86 (0.38–1.90)
Race/ethnicity Non-Hispanic White Non-Hispanic Black Hispanic Mexican American Asian Other	67% (46/76) 52% (41/83) 45.9% (6/15) 39.8% (8/18) 13.4% (1/5) 54.4% (6/9)	REF 0.51 (0.24–1.08) 0.42 (0.15–1.09) 0.33 (0.15–0.72)^a^ 0.07 (0.0007–0.79)^a^ 0.59 (0.1–3.5)
Level of education No high school diploma High school diploma Beyond high school diploma	69.3% (36/66) 56% (35/68) 59.2% (37/72)	REF 0.56 (0.27–1.18) 0.64 (0.18–2.25)
Annual family income, USD <25,000 25,000–55,000 >55,000	49.5% (36/71) 71.5% (16/26) 44.1% (6/13)	REF 2.62 (0.77–8.82) 0.81 (0.21–5.51)
Insurance No Yes	56.8% (27/58) 62.9% (81/148)	REF 1.23 (0.66–2.76)
Insurance type Medicare Medicaid Private Government Other	68.4% (18/30) 54.2% (28/54) 57.5% (23/41) 59.5% (7/12) 50.2% (5/11)	REF 0.76 (0.38–2.51) 0.85 (0.46–2.76) 0.88 (0.49–2.84) 0.67 (0.28–1.96)
Health care access No Yes	57.2% (20/44) 66.3% (88/162)	REF 1.60 (0.85–3.76)
Citizen^b^ US citizen Non-citizen	60.4% (61/111) 0% (0/6)	REF <0.001^a^^,^^c^
Birth cohort US-born Non–US-born	63% (103/185) 24.3% (5/21)	REF 0.19 (0.06–0.55)^a^

Abbreviations: HCV, hepatitis C virus; OR, odds ratio.

^a^ Significant value.

^b^ Data missing for 89 participants.

^c^OR could not be reported due to the numbers.

When looking at the trend of HCV infection awareness over the years (Table 2), there was an improvement from 2013 to 2018. January 2019 to March 2020 data were excluded in order to have three 2-year samples for better comparability. From 2013 to 2014, 54.2% of the population was estimated to be aware of their HCV infection, which increased to 60.5% in the 2017 to 2018 cycle; however, this difference did not reach statistical significance. There is still an estimated 0.86 million people who remained unaware in the 2017 to 2018 cycle. Those with HCV infection also had significantly higher liver enzymes than those without infection (Table 3). The mean (standard error [SE]) aspartate transferase level was 56.6 (4.1) U/L in the infected cohort, compared to 23.5 (0.14) U/L in the uninfected cohort, and mean (SE) alanine transaminase level was 60.3 (4.9) U/L compared to 23.8 (0.18) U/L. Among those with HCV infection, liver enzyme levels were higher in the group unaware of their infection status (Table 4). Mean (SE) alanine transaminase levels were significantly higher at 70.5 (7.2) U/L in those unaware of their infection compared to 53.9 (5.2) U/L in those who were aware (p = 0.041).

**Table 2:** Awareness of HCV antibody and HCV infection (HCV RNA), 2013–2018.

Years	HCV awareness weighted %	Active HCV, not aware of infection, No.	Adjusted OR (95% CI)
2013–14	54.2%	880,753	REF
2015–16	56.8%	888,567	1.18 (0.33–3.23)
2017–18^a^	60.5%	864,995	1.46 (0.52–4.15)

Abbreviations: HCV, hepatitis C virus; OR, odds ratio; REF, reference.

^a^ 2017–2018 used instead of 2017–2020 for better comparability in 2-year periods.

**Table 3:** Liver function tests among those with and without active HCV^a^.

Liver function test	Not infected	HCV infected	*P* value
AST, mean (SE), U/L	23.5 (0.14)	56.6 (4.1)	<0.0001^b^
ALT, mean (SE), U/L	23.8 (0.18)	60.3 (4.9)	<0.0001^b^
ALP, mean (SE), IU/L	70.5 (0.36)	78 (1.9)	0.002^b^
Total bilirubin, mean (SE), mg/dL	0.54 (0.005)	0.55 (0.03)	0.70
Serum albumin, mean (SE), g/dL	4.2 (0.007)	4.04 (0.04)	0.001^b^

Abbreviations: ALT, alanine transaminase; ALP, alkaline phosphatase; AST, aspartate transferase; coeff, coefficient; HCV, hepatitis C virus; REF, reference; SE, standard error.

^a^ Participants with positive HCV RNA are identified as HCV infected.

^b^ Significant value.

**Table 4:** Liver function tests among those aware and unaware of their active HCV infection^a^.

Liver function test	Active HCV, aware of infection	Active HCV, not aware of infection	*P* value
AST, mean (SE), U/L	53.7 (5.2)	61.2 (4.2)	0.24
ALT, mean (SE), U/L	53.9 (5.2)	70.5 (7.2)	0.041^b^
ALP, mean (SE), IU/L	79.0 (2.9)	76.3 (2.6)	0.54
Total bilirubin, mean (SE), mg/dL	0.54 (0.03)	0.58 (0.04)	0.51
Serum albumin, mean (SE), g/dL	4.0 (0.05)	4.1 (0.06)	0.12

Abbreviations: ALT, alanine transaminase; ALP, alkaline phosphatase; AST, aspartate transferase; coeff, coefficient; HCV, hepatitis C virus; REF, reference; SE, standard error.

^a^ Participants with positive HCV RNA are identified as HCV infected.

^b^ Significant value.

## Discussion

The most well-known barrier to the treatment of HCV and, ultimately, its elimination as a public health threat is the large number of undiagnosed cases [18] Awareness of HCV infection in the US population was estimated at 49% from 2007 to 2008 [18] Although we have seen a gradual increase in awareness since then and from 2013 through 2018, it remains suboptimal. Based on our study, almost 40% of the US population remains unaware of their HCV infection. Despite the improvement in the percentage of people aware of their infection status, the absolute number of patients unaware of their illness has not changed much. This is due to a concomitant increase in the number of people living with HCV. Although the cure rates and sustained virologic response are phenomenal with DAA therapy, it is vital to diagnose the cases first. These are data before the COVID-19 pandemic, which has resulted in widespread disruption ofroutine health care and screening. The pandemic resulted in significant reductions in testing volumes, and a 40–50% reduction in new referrals and treatment in 2020–2021 across various parts of the world [19,20]. Since HCV tends to be asymptomatic, more aggressive measures in the form of public health messaging and outreach camps for screening, diagnosis, and referrals are paramount. Early diagnosis of these asymptomatic patients will help initiate treatment in patients at early stages of fibrosis. This has been shown to have better sustained virological response and outcomes [21].

According to CDC reports, HCV infection has been traditionally low in those younger than 20 and older than 60 years of age. Our study showed that adults between 36 and 50 years had a lower awareness (46.1%) than other age groups. Diagnosing these patients is important as the risk of complications and comorbidities can rise as they age. The racial and ethnic disparity in knowledge of HCV has been shown in earlier studies [11,12]. Race and ethnicity also impact the natural history of HCV, with Hispanics having higher risk of cirrhosis [22], largely attributed to an earlier age at infection and higher prevelance of coexisting nonalcoholic fatty liver disease. The Black population has a lower risk of cirrhosis but higher mortality and complication rates once cirrhosis develops [23]. Racial and ethnic minorities have been shown to have a lower treatment ratio than White patients after adjusting for insurance status [24]. Our study also indicates that awareness was lowest in Asian and Mexican American populations, and Hispanic and Black populations had lower awareness than the White population. Prior studies have also shown that individuals of Asian descent are less likely to have primary care physicians or see a physician for chronic illness and preventative medicine compared to people of other races [25]. These results reiterate the importance of focused efforts toward racial and ethnic minorities with chronic HCV infection to improve their access to screening and treatment.

As shown, non-US citizens and non–US-born individuals are significantly more likely to have undiagnosed HCVinfection. Immigrants account for a disproportionately large number of HCV cases in North America and have worse outcomes, including cirrhosis and hepatocellular carcinoma [26]. Coming from areas of high endemicity, insufficient knowledge, and poor access to health care all contribute to their higher risk of delayed diagnosis. Early screening programs and linkage to care for immigrant people from countries with a higher prevalence of HCV would go a long way towards treating the disease in this population and are also vital to achieving HCV elimination. Although a generalized approach has been shown to be effective in the screening and treatment of HCV, clinicians must recognize high-risk populations and the unique challenges in each group.

The CDC recommends universal screening for HCV [10] for all adults 18 years and older, and during each pregnancy except in areas where the prevalence of the infection is less than 0.1%. Regular screening is encouraged in high-risk groups irrespective of age and prevalence. Our results also show that liver transaminases are significantly higher in participants with confirmed HCV compared to those who are uninfected, which is an expected finding. Although there is no direct correlation between transaminases and fibrosis grade, elevated LFTs in chronic HCV infection indicate ongoing inflammation and the severity of liver damage [27,28] We also see that the amplitude of transaminase elevation is higher in the undiagnosed population; this group has a higher risk of hepatic necrosis and fibrosis progression. Elevated transaminase levels should prompt screening for HCV infection, especially in places with limited resources or access to health services and where routine screening has not been implemented.

A limitation of our study, as inherent to most NHANES-based studies, is that this is only a sampling of the US population. The results may not be generalizable globally, but this public health issue is likely of higher amplitude in many other parts of the world. Several high-risk groups for HCV, including institutionalized, homeless, and those in nursing homes, are not represented. Hence, this might underrepresent the burden of disease, the number of those with HCV infection, and those who remain undiagnosed and untreated.

### Conclusion

Our results reveal that an estimated 39.9% (approximately 0.84 million) of individuals with HCV in the US are unaware of their illness. Although the proportion of people aware of the infection has improved with time, the absolute numbers have remained high due to the increasing number of people living with HCV infection. With advancements in HCV treatment, it is essential to diagnose these cases early to avoid liver damage and progress toward the WHO goal of eliminating the disease. Racial minorities, especially Asian and Mexican American populations, non-US citizens, and non–US-born adults, are at high risk of having undiagnosed HCV infection. Future studies and projects directed toward underprivileged and high-risk populations might help understand and eliminate barriers specific to these populations.
